# Sexual Activity during Menstruation in The Holy Bible and Quran

**DOI:** 10.22074/ijfs.2020.6060

**Published:** 2020-02-25

**Authors:** Elias E. Mazokopakis

**Affiliations:** 1 Department of Internal Medicine, Naval Hospital of Crete, Chania, Greece; 2 Department of Theology, School of Theology, National and Kapodistrian University of Athens, Athens, Greece

I read with great interest the manuscript entitled "Association between sexual activity during menstruation and endometriosis: a case-control study" published in the International Journal of Fertility and Sterility (1). According to the findings of this study, there was an association between sexual activities leading to orgasm during menstruation and endometriosis (1). These findings revealed the medical value and health benefit of the restriction of sexual contact during menstruation by major religions of the world. Particularly, in the third Book of the Pentateuch or Torah, known as Leviticus, it states that a woman undergoing menstruation is perceived as unclean for seven days and whoever touches her shall be unclean until evening (Leviticus 15:19). Moreover, “if a man actually lies with her so that her menstrual impurity is on him, he shall be unclean seven days, and every bed on which he lies shall be unclean” (Leviticus 15:24). In other words, if a man has sexual relations with a menstruating woman, he is not perceived as unclean only until evening, but for seven days. When seven days pass from the beginning of menstruation, the woman is regarded as clean and thus sexual contact is permitted. In another biblical passage it is stated that if a man has sexual relations with a woman during her menstrual period, both of them must be cut off from the community for they have both been exposed the source of her blood flow (Leviticus 20:18). The same biblical attitude to sexual relations during menstruation is also described in the following biblical passages: Leviticus 18:19 and 12:2, Ezekiel 18:5, 18:6, 18:9 and 22:10. These biblical sources were the reason for the attitudes of Judaism, as were the attitudes of subsequent religions that were influenced by Judaism, namely Christianity and Islam (2). In Christianity, sexual contact that occurs during menstruation is also considered as prostitution, since its unique purpose is usually the satisfaction of (man’s mainly) sexual instinct and the achievement of pleasure (2). In addition to Judaism and Christianity, Islam also forbids men to have vaginal sexual intercourse with their wives during menstruation (Surah al-Baqarah 2:222). Other risks from sexual intercourse with a menstruating woman, except endometriosis, include the development of sexually transmitted diseases, increase in the flow of menstrual blood, and an undesirable pregnancy (2).
